# Molecular Atlas of HER2+ Breast Cancer Cells Treated with Endogenous Ligands: Temporal Insights into Mechanisms of Trastuzumab Resistance

**DOI:** 10.3390/cancers16030553

**Published:** 2024-01-27

**Authors:** Kavitha Mukund, Jackelyn A. Alva-Ornelas, Adam L. Maddox, Divya Murali, Darya Veraksa, Andras Saftics, Jerneja Tomsic, David Frankhouser, Meagan Razo, Tijana Jovanovic-Talisman, Victoria L. Seewaldt, Shankar Subramaniam

**Affiliations:** 1 Department of Bioengineering, UC San Diego, Gilman Drive, La Jolla, CA 92093, USA; k1mukund@ucsd.edu (K.M.); dimurali@ucsd.edu (D.M.); dveraksa@ucsd.edu (D.V.); 2 City of Hope Comprehensive Cancer Center, 1500 East Duarte Road, Duarte, CA 91010, USA; jalvao@coh.org (J.A.A.-O.); jtomsic@coh.org (J.T.); dfrankhouser@coh.org (D.F.); mrazo@coh.org (M.R.); 3 Department of Cancer Biology and Molecular Medicine, Beckman Research Institute, City of Hope, 1500 East Duarte Road, Duarte, CA 91010, USA; amaddox@coh.org (A.L.M.); asaftics@coh.org (A.S.); ttalisman@coh.org (T.J.-T.)

**Keywords:** HER2+, breast cancer, qSMLM, multiomics, trastuzumab resistance, endogenous ligands, EGF, HRG

## Abstract

**Simple Summary:**

A major hurdle in trastuzumab therapy remains acquired resistance within some HER2+ patients. Mechanisms contributing to, and as a consequence of, resistance remain elusive. Here, we investigate the spatiotemporal change in cell states within two cell lines—trastuzumab-sensitive (BT474) and -resistant (BT474R) HER2+ cancer cells in the presence of two endogenous ligands, epidermal growth factor (EGF) and neuregulin1/heregulin (HRG). We measure the HER2 receptor organization dynamics, signaling state, chromatin accessibility, and bioenergetics in both cell lines. Our measurements reveal significant alterations in a ligand-dependent manner within both cell lines. To the best of our knowledge, this study represents a novel attempt at systematically characterizing the effect of ligands on HER2 overexpressing cells, emphasizing their pivotal role. It provides critical insights into the dynamics of trastuzumab resistance in these cancers.

**Abstract:**

Trastuzumab therapy in HER2+ breast cancer patients has mixed success owing to acquired resistance to therapy. A detailed understanding of downstream molecular cascades resulting from trastuzumab resistance is yet to emerge. In this study, we investigate the cellular mechanisms underlying acquired resistance using trastuzumab-sensitive and -resistant cancer cells (BT474 and BT474R) treated with endogenous ligands EGF and HRG across time. We probe early receptor organization through microscopy and signaling events through multiomics measurements and assess the bioenergetic state through mitochondrial measurements. Integrative analyses of our measurements reveal significant alterations in EGF-treated BT474 HER2 membrane dynamics and robust downstream activation of PI3K/AKT/mTORC1 signaling. EGF-treated BT474R shows a sustained interferon-independent activation of the IRF1/STAT1 cascade, potentially contributing to trastuzumab resistance. Both cell lines exhibit temporally divergent metabolic demands and HIF1A-mediated stress responses. BT474R demonstrates inherently increased mitochondrial activity. HRG treatment in BT474R leads to a pronounced reduction in AR expression, affecting downstream lipid metabolism with implications for treatment response. Our results provide novel insights into mechanistic changes underlying ligand treatment in BT474 and BT474R and emphasize the pivotal role of endogenous ligands. These results can serve as a framework for furthering the understanding of trastuzumab resistance, with therapeutic implications for women with acquired resistance.

## 1. Introduction

The ERBB network is the dominant driver of aberrant cell signaling in a majority of HER2+ breast cancers. The ERBB network is comprised largely of four receptor tyrosine kinases (RTKs), namely, HER1 (*EGFR*), HER2 (*ERBB2*), HER3 (*ERBB3*), and HER4 (*ERBB4*) and their downstream signaling cascades [1]. In contrast to the other HER-family members, HER2 lacks a ligand and is activated in a ligand-independent manner either through homodimerization or heterodimerization with other HERs. For instance, HER1 can bind exclusively to ligands such as epidermal growth factor (EGF), while HER3 and HER4 can bind neuregulin1/heregulin (HRG) to promote dimerization with HER2 and stimulate downstream signaling [2]. Signaling downstream of activated HER2 [3] occurs through a network of highly interlinked signaling cascades, mainly PI3K/AKT and RAS/MAPK, affecting cell proliferation, survival, differentiation, angiogenesis, invasion, and metastasis [2,4]. Monoclonal antibodies commonly used in treating HER2+ breast cancers, including trastuzumab, work by binding the extracellular domain of HER2 and impact several aspects of HER2-driven signaling, including disruption of the receptor dimerization dynamics and inhibition of extracellular HER2 proteolytic cleavage, receptor internalization, and degradation. While HER2-directed trastuzumab therapy has significantly improved patient outcomes, a subset of patients acquire resistance and do not respond adequately. Several mechanisms have been proposed to confer resistance to HER2-directed therapy, including steric hindrance of the extracellular domain, epitope masking, mutations within the targeted receptor, and subsequent alterations in signaling pathways through activating mutations and crosstalk among the various receptor tyrosine kinases that interact with HER2 [5,6].

While the cause of resistance to HER2-directed therapy in breast cancer has been broadly characterized, as highlighted above, to the best of our knowledge, mechanisms downstream of acquired resistance have been less systematically analyzed. Understanding the molecular mechanisms and the downstream changes in resistant cells in the presence of endogenous ligands can shed light on approaches to understanding the consequences of trastuzumab resistance.

In this study, we characterize the molecular changes in HER2 overexpressing breast cancer cell lines that are sensitive (BT474 breast ductal carcinoma lineage cell lines) and resistant (BT474R) to anti-HER2 treatment when stimulated with two major ligands involved in HER2 signaling—EGF and HRG. We investigate their responses across time (30 min, 1 h, 12 h, and 24 h) using multiple measurement modalities. Specifically, we quantify membrane levels of HER2 receptors using quantitative single-molecule localization microscopy (qSMLM) and changes in downstream signaling key nodes using reverse phase protein assays (RPPA), RNA, and assay for transposase-accessible chromatin sequencing (RNAseq and ATACseq). In the first section of the results, we outline the experimental design and data analysis strategies adopted in this study. In the sections following this, we highlight the major mechanistic differences that exist in BT474 and BT474R after treatment with ligands EGF and HRG. Specifically, we explore the differences in HER2 receptor density and clustering within these cell lines, followed by an analysis of the immediate downstream signaling changes as inferred from the multiomics measurements. Alterations in bioenergetics (glycolytic and mitochondrial) in both cell lines are also presented. Cell line-specific alterations are explored in greater detail, including the dysregulation of AR and its likely impact on lipid metabolism within HRG-treated BT474R (henceforth referred to as HRG-BT474R). We also investigated the role of interferon regulatory factor-1 signaling exclusively in EGF-treated BT474R (henceforth referred to as EGF-BT474R). Given the presence of a hypoxic response in both EGF-treated BT474 (EGF-BT474) and EGF-BT474R, we present a more nuanced understanding of the functional differences that exist between the cell lines using a differential co-expression network approach. Finally, we present a unified and detailed mechanistic view of the effect of ligand treatment on trastuzumab-resistant and -sensitive cell lines and its possible clinical implications. All the data generated in this study are available through the Gene Expression Omnibus with accession GSE237607, and the processed data are accessible via https://www.tnbcworkbench.org/shiny/BT474_RShiny/.

## 2. Materials and Methods

### 2.1. Single-Molecule Localization Microscopy (SMLM)

#### 2.1.1. Cell Culture and Ligand Treatments for SMLM

The BT474 cell line and a BT474 clone 5 (BT474R) originally generated from the BT474 cell line grown in the presence of 10 μg/mL trastuzumab for several weeks [7] (American Type Culture Collection, Manassas, VA, USA) were maintained at 37 °C and 5% CO_2_ in phenol red-free DMEM (Invitrogen, Waltham, MA, USA) supplemented with 10% heat-inactivated fetal bovine serum (FBS, VWR International, Radnor, PA, USA), 4 mM of Glutamax (Invitrogen) and 1 mM of sodium pyruvate (Invitrogen). Cell lines were passaged using TrypLE Select Enzyme (Invitrogen). Triple-negative breast cancer cell line, MDA-MB-468 cell line (American Type Culture Collection), was cultured at 37 °C and 5% CO_2_ in phenol red-free DMEM supplemented with 10% FBS, 1 mM of sodium pyruvate, and 4 mM of GlutaMAX. For SMLM, BT474, BT474R, and MDA-MB-468 cells were seeded on clean coverslips for 36 h and switched to starvation media (phenol red-free DMEM supplemented with 0.1% FBS, 1 mM of sodium pyruvate, and 4 mM of GlutaMAX) for 14 h. For BT474 and BT474R cells, the media was aspirated, and fresh starvation media containing specific ligands were added and placed in the cell culture incubator for 30 min, 1 h, and 12 h. Concentrations used for ligand experiments were 50 ng/mL for EGF (GenScript Biotech, Piscataway, NJ, USA) and 10 ng/mL for HRG (GenScript Biotech). Cells were stained with fluorescent antibodies (trastuzumab) and fixed as described before [8].

#### 2.1.2. Characterizing Photoswitching Behavior of Fluorescently Labeled Antibodies

To detect HER2, trastuzumab (Genentech, South San Francisco, CA, USA) was labeled with the AF647 dye (Invitrogen). Following an optimized protocol for dye conjugation, each antibody was labeled with approximately one fluorescent dye (degree of labeling (DOL)~1). In SMLM, fluorescent probes turn “on” and “off”, and localization of these probes in “on” states is detected. Typically, each probe appears in several (not necessarily subsequent) “on” frames. To enable robust data quantification, we need to determine the average number of appearances of fluorescent probes (the average number of “on” frames) and maximal dark time (maximal time a probe can be in the “off” state). It was critical to define these parameters using identical experimental, optical, and imaging conditions to those used for cell experiments. Since MDA-MB-468 cells have very low HER2 density (receptors are well separated) [9], we used these cells under starved conditions to determine the photophysical properties of individual, fluorescently labeled trastuzumab. Cells were stained and imaged, and data were analyzed as described before [9].

#### 2.1.3. Qualitative Data Analysis and Statistical Considerations

Imaging setup, SMLM imaging, and quantitative data analysis of cells were performed as described before [8]. Briefly, to determine the detected densities of HER2, the total number of localizations acquired during image acquisition was divided by the average number of appearances for the fluorescent probe (trastuzumab-AF467). The average number of appearances combined with pair-correlation (PC) analysis was used to determine HER2 cluster radius and number of HER2 molecules per cluster for regions of interest (ROIs) with non-random HER2 organization. For the same ROIs, the maximum dark time, average number of appearances, values for cluster size (from PC analysis), and a k-means-like clustering algorithm were used to determine the number of HER2 clusters with more than two molecules and the fraction of clustered HER2 molecules relative to all detected HER2 molecules.

Statistical Considerations. A minimum of 13 cells were imaged for ligand treatments in BT474 and BT474R, except for EGF treatment at 1 h and 12 h time points, which had a minimum of 10 cells. Sufficient cell sampling for each condition was evaluated using *p*-value_split_ as described before [9]; for all measured variables in both cell lines, this value was above 0.1. *p* values were calculated in Excel using a one-tailed Student’s *t*-test with a heteroscedastic two-sample unequal variance type.

### 2.2. Multiomics Measurements and Data Processing

#### 2.2.1. Cell Culture and Ligand Treatment for Omics Data

For ligand treatments, 500,000 BT474 and BT474R cells/well were plated in 6-well plates and cultured for 48 h in complete media with one media change after 24 h. Next, cells were serum starved using starvation media for 14–16 h. After serum starvation, cells were treated with 50 ng/mL of EGF, 10 ng/mL HRG, or vehicle (water) in freshly made serum starvation media for 30 min, 1 h, 12 h, or 24 h. Time-zero (T0) samples were collected after serum starvation. All treatments were carried out in duplicate. All cell lines in this study were (1) validated for authenticity and (2) tested for mycoplasma by the City of Hope Analytical Pharmacology Core Shared Resource; mycoplasma testing was performed at the outset of experiments and repeated monthly. Cell lines were maintained and treated in the absence of antibiotics; all experiments were passaged, matched, and controlled for confluency.

#### 2.2.2. Reverse Phase Protein Arrays

Treated BT474 and BT474R were washed in cold phosphate-buffered saline (PBS) and lysed using 150 μL of lysis buffer (provided by the RPPA core) and incubated on ice for 20 min with shaking every 5 min. Cell lysates were centrifuged at 14,000 rpm for 10 min at 4 °C. Protein concentration was determined using the Pierce BCA Protein Assay kit (Waltham, MA, USA). Protein concentration was adjusted to 1.5 mg/mL in 4× SDS sample buffer (provided by RPPA core) containing 2-mercaptoethanol (Sigma, St. Louis, MO, USA) and boiled for 5 min. Samples for RPPA were processed through the Functional Proteomic Reverse Phase Protein Array Core at the MD Anderson Cancer Center (MDACC) at the University of Texas. Samples were serially diluted two-fold for 5 dilutions (undiluted, 1:2, 1:4, 1:8; 1:16) and arrayed on nitrocellulose-coated slides (Grace Bio-labs, Bend, OR, USA) by the Quanterix (Aushon) 2470 Arrayer (Quanterix Corporation, Billerica, MA, USA) in an 11 × 11 format to produce sample spots. Sample spots were then probed with antibodies using a tyramide-based signal amplification approach and visualized by DAB colorimetric reaction to produce stained slides. Stained slides were scanned on a Huron TissueScope scanner (Huron Digital Pathology, St. Jacobs, ON, Canada) to produce 16-bit tiff images. Sample spots in tiff images were identified, and their densities were quantified by Array-Pro Analyzer (Media Cybernetics, Rockville, MD, USA). RPPA slides were stained for 485 unique antibodies, which were analyzed by Array-Pro Analyzer 6.3, then by SuperCurve_1.5.0 via SuperCurveGUI_2.1.1. Quality control (QC) tests were performed for each antibody staining (slide).

#### 2.2.3. RNA-Sequencing

Treated BT474 and BT474R were collected using TrypLE Select and washed using PBS. RNA extraction was performed using an RNeasy Mini Kit (Qiagen, Hilden, Germany), including on-column DNase digestion using RNase-free DNase (Qiagen). Sample QC and library preparation were performed by the Integrative Genomics Core (IGC) Shared Resource at the City of Hope. RNA sequencing libraries were prepared with Kapa RNA mRNA HyperPrep kit (Kapa Biosystems, Wilmington, MA, USA) according to the manufacturer’s protocol. The libraries were validated with the Bioanalyzer DNA High Sensitivity Kit (Agilent, Santa Clara, CA, USA) and quantified with Qubit Fluorometer (Invitrogen). Sequencing was performed on Illumina NovaSeq6000 with S4 Reagent v1.5 kit (Illumina, San Diego, CA, USA) at the Translational Genomics Research Institute (TGen) with the sequencing length of 2 × 101 (100 bp, paired-end reads) with an average of ~ 34.8 million reads per sample. Real-time analysis (RTA) 3.4.4 software was used to process the image analysis.

#### 2.2.4. Assay for Transposase-Accessible Chromatin (ATAC)-Sequencing

ATAC was performed on treated BT474 and BT474R cells based on the Omni-ATAC protocol [10]. Briefly, 50,000 single cells were lysed using cell lysis buffer (0.1% NP-40 (Sigma), 0.1% tween-20 (Sigma), and 0.01% digitonin (Promega, Madison, WI, USA) in resuspension buffer (10 mM of Tris-HCl pH 7.5, 10 mM of NaCl, and 3 mM of MgCl_2_ in water; all chemicals were purchased from Sigma) for 3 min on ice. After washing using resuspension buffer containing 0.1% tween-20, cells were centrifuged at 500× *g* for 10 min at 4 °C. The transposition reaction was performed using Tagment DNA Enzyme and buffer (Illumina) at 37 °C for 30 min in an Eppendorf (Hamburg, Germany) ThermoMixer (1000 rpm). DNA was purified using the MinElute Reaction Cleanup kit (Qiagen). Libraries were prepared by amplifying DNA for 5–9 cycles using DNA/RNA UD Indexes Set A (Illumina) and NEBNext High-Fidelity 2X PCR Master Mix (New England Biolabs, Ipswich, MA, USA). AMPure XP bead purification (Beckman Coulter, Brea, CA, USA) was used for the PCR product cleanup. The libraries were validated with Agilent Bioanalyzer DNA High Sensitivity kit and quantified with qPCR at the IGC. ATAC-seq libraries were sequenced on Illumina NovaSeq6000 with S4 Reagent v1.5 kit (Illumina) at TGen with the sequencing length of 2 × 101. Real-time analysis (RTA) 3.4.4 software was used to process the image analysis.

### 2.3. Mitotracker, Glucose Uptake, and Intracellular Calcium Assays

Mitotracker assay—After treatments, 250,000 BT474 and BT474R cells were stained with 2 μM of JC-1 (MitoProbe JC-1 Assay kit for flow cytometry, Invitrogen) in media containing 0.1% FBS for 15 min at 37 °C. Cells were washed with cold PBS containing 1% FBS and analyzed on a Becton Dickinson (BD, Franklin Lakes, NJ, USA) LSRFortessa Cell Analyzer. Data were processed using BD FACSDiva software v 8.0.2.

Glucose uptake assay—After treatment, 300,000 BT474 and BT474R cells were incubated with 100 μM of 2-NBDG (Invitrogen) in DMEM (no glucose) containing 0.1% FBS for 30 min at 37 °C. Cells were washed with cold PBS containing 1% FBS and resuspended in wash buffer containing 0.3 μg/mL of DAPI (Invitrogen). Samples were analyzed on a BD LSRFortessa Cell Analyzer. Data were processed using BD FACSDiva software.

Intracellular calcium assay—After treatment, BT474 and BT474R cells were plated in a 96-well plate (20,000 cells/well) and loaded with Fluo-4 Direct from the Calcium Assay kit (Invitrogen) for 1 h at 37 °C. Fluorescence was measured at 494 nm using a Cytation 3 plate reader (Biotek, Winooski, VT, USA).

### 2.4. Multiomic Data Processing and Analyses

#### 2.4.1. RPPA Data Processing

Our samples with HRG, vehicle/water treatment, and baseline (T0) and EGF treatments were processed in different batches at different times with slightly differing antibody panels and controls (472 and 485 antibodies (Ab), respectively) using the MDACC Functional Proteomic Reverse Phase Protein Array Core. Subsequently, the RPPA data were de-batched, normalized for protein loading, and transformed to log2 values in a suitable manner as per MDACC recommendations (personal communication), resulting in a common set of 465 Ab, which was used for all downstream analyses. Significance to call differentially expressed proteins (DEPs, *p* < 0.05) for all pairwise comparisons was performed using Welch’s *t*-test, as previously recommended. Of note, differential analysis for all the data modalities considered within the study, including RPPA, was performed at every time point for each treatment with respect to their respective vehicle/water controls, allowing delineation of the effect of ligand at a given time point.

#### 2.4.2. RNAseq Data Processing

The data for 40 samples (vehicle/water treated and 2 ligands treated—EGF and HRG; in duplicate x three time points (1 h, 12 h, and 24 h) and 2 replicates for T0 for BT474 and BT474R), with an average of 34.8 million reads, were processed at the UCSD Institute for Genomic Medicine (IGM) Genomics Center. Quality testing was performed using *Fastqc v0.11.8* (https://www.bioinformatics.babraham.ac.uk/projects/fastqc) and summarized for downstream analysis using *multiQC* (https://github.com/MultiQC/MultiQC). Trimming for Illumina universal adaptors was performed using *TrimGalore v0.6.6* (https://www.bioinformatics.babraham.ac.uk/projects/trim_galore/). RNA read mapping and alignment were performed on human reference genome GrCh38.p13 using *Rsubread v2.6.4* [11]. All multiomics data were processed using R v4.1.0/Bioconductor v3.13). The mapped reads were summarized to gene level using “featureCounts” of the *Rsubread* package and annotated corresponding to the reference genome. Count matrix normalization and differential analysis were performed using the *DEseq2 v1.32* package available through R/Bioconductor, as outlined in their vignette [12]. DESeq models count data as negative binomial distribution, with variance and mean linked by local regression. *DESeq2* builds DESeq by utilizing shrinkage estimates for dispersions and fold changes, improving stability and interpretability. Based on the principal component analysis of the 40-sample matrix and *DESeq2* recommendations, we split the matrix into resistant and sensitive and processed it individually for all downstream analyses. Genes with at least 1 count in at least half the samples were retained for further analysis within each matrix. The resulting count matrix used for differential gene analysis in *DESeq2* contained 22299 genes × 20 samples for BT474 and 21,689 genes × 20 samples for BT474R. The pairwise comparisons for each treatment at each time point were performed with respect to their respective vehicle/water controls at every time point. DEGs were called at a Benjamini–Hochberg false adjusted *p*  <  0.05. Heatmaps of fold changes were generated using *pheatmap v1.0.12* library in R [13].

#### 2.4.3. ATACseq Data Processing

ATAC sequencing was also performed using the 40 samples outlined above. Quality control of sequenced fastq files, using *Fastqc v0.11.8*, indicated Nextera adaptor content and were subsequently processed and cleaned using *TrimGalore*. The cleaned sequences were then mapped to the most current version of National Center for Biotechnology Information (NCBI) to human reference genome GrCh38.p13 using *BBMap*, available through the *BBTools* suite (https://sourceforge.net/projects/bbmap/) [14]. The mapped reads were then sorted and indexed using *samtools* (https://github.com/samtools/samtools) o allow for the use of *Genrich v0.6* peak caller (https://github.com/jsh58/Genrich). In addition to peak calling, *Genrich* was used on the sorted files to remove mitochondrial reads and reads mapping to ENCODE black-listed regions. Finally, differential peak analysis was performed using *DiffBind v3.2.7* [15] on the narrowPeak files generated by *Genrich*. *ChipSeeker v1.28.3* [16] was used to visualize and annotate peaks and perform all downstream analyses. *DiffTF v1.7* was utilized to calculate the differential transcription factor (TF) activity between two or more conditions for each TF by comparing the distribution of fold-change differences across all binding sites of a TF to all binding sites from all other TFs. The algorithm was used as described in [17]. Enhancers were identified using the PEREGRINE database (https://www.peregrineproj.org, last accessed 3 March 2022), with only breast/breast-derived cells as tissue of interest [18]. All the data generated in this study are available through the Gene Expression Omnibus with accession GSE237607.

#### 2.4.4. RShiny App 

An interactive visualization of the processed data from RPPA, RNAseq, and ATACseq data was developed using the *Shiny v1.7.5* library in R. It is currently hosted and accessible via https://www.tnbcworkbench.org/shiny/BT474_RShiny/. For each of the data modalities processed in this study, the user is able to explore and visualize the data in several ways. For RPPA, expression heatmaps of log2 transformed RPPA data for the available 465 Ab were created using the *ComplexHeatmap v2.18.0* library [19]. The user can also visualize the DEPs for each pairwise comparison of conditions based on custom *p*-value and log2 fold change thresholds. Likewise, the user can visualize DEGs for each pairwise comparison of conditions based on custom thresholds from RNAseq data. Other RNAseq visualizations include boxplots of rlog normalized expression across 2 replicates for each gene, heatmaps of average rlog normalized expression for user-selected genes, and heatmaps of log2 fold changes between vehicle/water controls and each condition for user-selected genes. Finally, for ATACseq data, the user can visualize average peak profile plots for each condition, which is implemented using the *ChIPseeker v1.32*, enhancer binding sites that regulate each gene using PEREGRINE and *chromoMap v4.1.1* [20], and open binding sites for each condition across 2 replicates using *Gviz v1.42.1* [21]. All visualizations include the capability to save the plots and download the data that were used to generate them.

### 2.5. Network Clustering and Analysis

A network of very high confidence (>0.85) protein–protein interactions (PPI) for humans (TXID 9606) was downloaded from the STRING database (v11, last accessed June 2020) [22], containing 12,402 proteins and 139,384 interactions. We also created a custom TF-target network by combining three TF-target data, namely, the A and B confidence TF-target interactions derived from *DoRothEA v1.0* and the TRANSFAC 2019 database (release 2019.3, https://genexplain.com/transfac/, last accessed 2019), and the TF-target interactions from [23]. The resulting TF-target network consisted of 7369 TFs and targets with 30,933 interactions, which was merged with the Human String PPI derived above, resulting in a PPI-TF-target network comprising 14,738 nodes with 169,711 edges. EGF and HRG ligand treatment-specific TF-target/PPI networks were extracted using the superset of DEGs in BT474 and BT474R, which were subsequently used for all downstream analyses. For BT474R, we had two TF-target/PPI networks corresponding to EGF and HRG ligands treatment with 3466 nodes and 18,131 edges and 1287 nodes and 4446 edges, respectively. We next extracted a subnetwork of proteins/genes that were the 1-step neighborhood of IRF1 and had some expression quantification in our RNAseq data. These genes were then used for downstream analysis using the “degPatterns” available through *DEGreport v1.32.0* [24].

### 2.6. Functional Annotation and Enrichment

Functional enrichment was identified through functional annotation clustering available through *clusterProfiler v4.0.5* [25] or *Enrichr v2.1* [26] in R/BioC. Gene ontology (biological process and molecular function) and KEGG pathway analyses were all performed within *clusterProfiler* using “EnrichKEGG” and “EnrichGO” functions. Likewise, Hallmark database was accessed through R’s *msigdbR v7.1.1,* and enrichment was assessed using “Enricher” function in *clusterProfiler*. Enrichment terms ranked by their *p*-value are presented for all comparative analysis.

### 2.7. Differential Correlation Network Analysis

We computed a differential correlation network for each treatment between the two cell lines. That is, the correlation coefficients were computed on variance stabilized (vst) normalized gene expression (across time) after EGF treatment for defined marker list (see above) in BT474R (rR) and BT474 (rS). These correlation values were transformed— namely, rR and rS were transformed into zR and zS, respectively, based on Fisher’s transformation: $z=0.5\left(\log\left(\frac{1+r}{1-r}\right)\right)$.

Differences between the two correlations were computed using the following equation:

(1) $$\delta =\frac{\mathrm{zR}-\;\mathrm{zS}}{\sqrt{\frac{1}{\left(\mathrm{nR}-3\right)}+\frac{1}{\left(\mathrm{nS}-3\right)}}\;}$$

where nR and nS = 6 (2 samples × 3 time points for each treatment/cell line). Significance of differential correlations was established using the “comp.2.cc.fdr” function implemented within the *diffcorr v0.7 package* [27], which utilized the local false discovery rate (lfdr) derived from the *fdrtool v1.2.16* package [28] and was used for controlling true estimates and identifying significant differential correlations. Edges with a lfdr < 0.05 were considered for downstream analysis. Clustering of the differential correlation network was performed using the topological overlap measure computed on the scaled difference matrix delta using hierarchical clustering. Degree is a node-level parameter and has been previously defined for unweighted, signed networks as the difference between the total number of positive edges and the total number of negative edges incident upon a node. We extended this definition to weighted and signed networks, with edge weights [−1, 1] and defined signed connectivity (kWithin) for each node u, within a module M, as

(2) $$\mathrm{kWithin}\left(u\right)={\;\mathrm{conn}}^{+}\left(u\right)-{\;\mathrm{conn}}^{-}\left(u\right)\in M$$

where conn^+^ is the sum of all positive edges and conn^−^ is the absolute sum of all negative edges. This is akin to the softConnectivity metric available via the *WGCNA v1.70* package in R [29], albeit with a power = 1 and signed adjacency. The clustered differential network, with only significant differential correlations (lfdr < 0.05), was visualized in Cytoscape.

## 3. Results

### 3.1. Study Design

**Figure 1:**
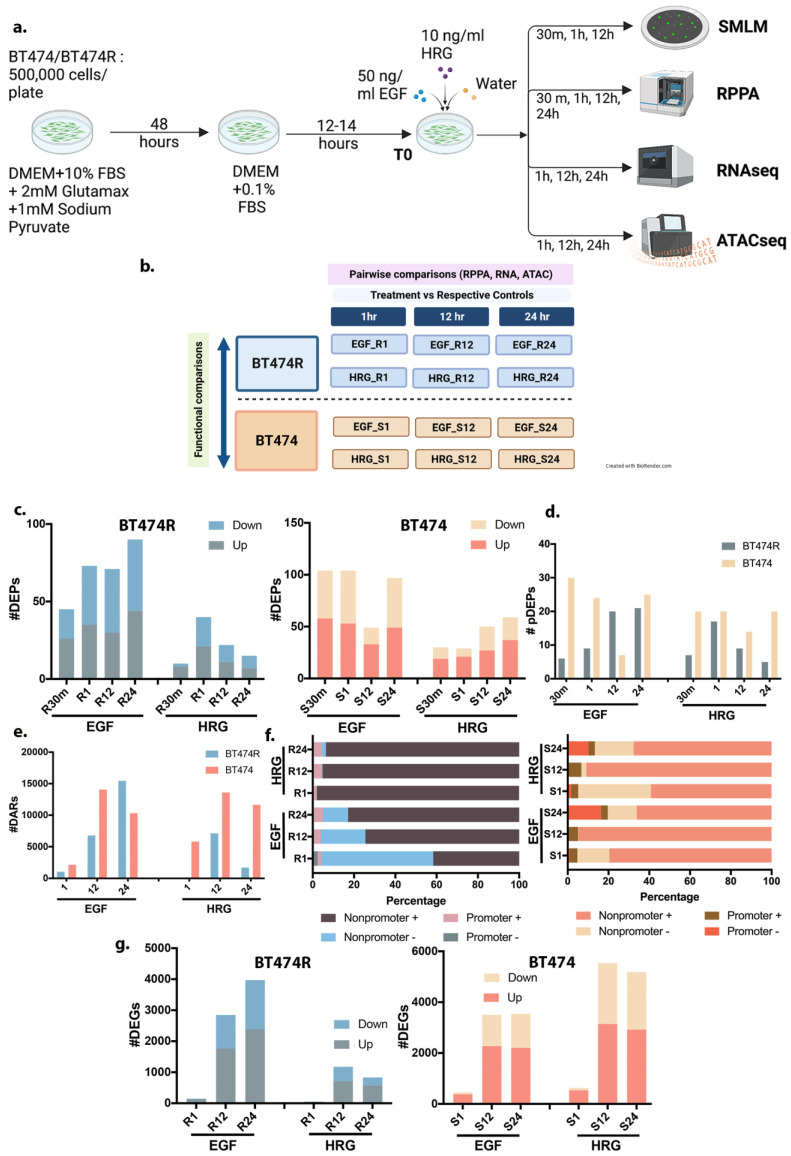
Study design and summary of multiomic measurements. (**a**) Study design—500,000 cells/well were plated in 6-well plates and cultured for 48 h in complete media with one media change after 24 h. Cells were serum starved using media containing 0.1% FBS for 14–16 h. After serum starvation, cells were treated with 50 ng/mL of epidermal growth factor (EGF), 10 ng/mL of heregulin (HRG), or vehicle (water) in freshly made serum starvation media for 30 min (for RPPA), 1 h, 12 h, or 24 h (for ATAC/RNAseq). Time-zero (T0) samples were collected after serum starvation. All treatments were performed in duplicate. (**b**) An infographic that highlights the types of comparisons performed within this manuscript. Differential expression (fold change) for each omics data modality (RNAseq, ATACseq, and RPPA) was computed as pairwise comparisons between treatment and their respective control for each time point. The results were then compared between the two cell lines to identify functional differences (functional comparisons). (**c**) Differentially expressed proteins (DEPs, adj *p* < 0.05) detected from RPPA measurements for 30 min, 1 h, 12 h, and 24 h for BT474R (R30, R1, R12, and R24, left panel) and sensitive cell line BT474 (S30, S1, S12, and S24, right panel) showed distinct and increased response in BT474 after treatment in contrast to BT474R. (**d**) A comparison of phosphorylated DEPs, which alludes to a highly active cell state, shows a more robust response in BT474 after both HRG and EGF. (**e**) A comparison of the differentially accessible regions (DARs) in BT474 and BT474R assessed from the ATACseq data reflected trends similar to DEPs, with BT474 overall being more accessible across time after treatments. (**f**) The proportion of DARs (as percentage of total DARs) that were observed in the promoter (upstream 1 kb downstream 1 kb of transcription start site (TSS)) and non-promoter regions of annotated genes (adj *p* < 0.05) for BT474R (left panel) and BT474 (right panel). BT474 clearly showed increased accessibility in its promoter region (compared to their respective control, “+”), especially after EGF. (**g**) Differentially expressed genes (*p* adj < 0.05) identified from RNAseq data reflected trends observed in RPPA and ATACseq with both EGF and HRG treated BT474 (right panel) in a transcriptionally more active state compared to BT474R (left panel) across time. Of note, BT474 seems to be most sensitive to treatment at 12 h, as observed.

We performed qSMLM, RPPA, RNA, and ATAC sequencing to dynamically interrogate the cell state as defined by HER2 receptor localization, downstream protein signaling, its transcriptomic landscape, and the accessibility of chromatin in response to ligands in BT474 and BT474R cultured cell lines. To capture the temporal dynamics of ligand treatments, samples were sequenced at four time points: T0 (baseline, pretreatment only), at 1 h, 12 h, and 24 h. Vehicle/water-treated cells at each of the same time points were used as their respective controls (Figure 1a). Protein lysates were additionally collected at 30 min for RPPA measurements to capture the phosphorylated protein dynamics. All samples for the above measurements were obtained in duplicates (n = 2), and differential analyses were performed with respect to controls at a given time point. Given the time complexity of qSMLM experiments, T0 was used as baseline control (n = 2–3). Sample preparation, extraction, and imaging/sequencing protocols for each of the aforementioned are presented in detail within the Section 2. Ligand concentrations were based on published studies [1,2,3,4,5] and titration experiments.

### 3.2. Processing of Multiomic Measurements from RPPA and RNA- and ATAC-Sequencing

Figure 1b encapsulates the analysis strategy adopted in this study for each multiomics data modality. Specifically, differential comparisons were performed pairwise, i.e., for each treatment compared to its respective control, at every time point, for BT474 and BT474R independently. These results were then used to compare and highlight differences at a functional/mechanistic level between BT474 and BT474R.

RPPA antibody measurements assess both modified and unmodified proteins within the cell. We utilized a common list of 465 antibodies used in RPPA measurements (see Section 2 for details) across the two cell lines for all downstream processing. Differentially expressed proteins and phosphoproteins (DEPs and pDEPs) were identified for each treatment condition (EGF and HRG) with respect to their vehicle/water treated controls at every time point (Figure 1c). Fold changes of DEPs for each ligand are reported in Table S1. The response of BT474R and BT474 to EGF was more robust than to HRG. HRG-BT474R showed only a modest perturbation at the protein level, which reduces across time in contrast to HRG-BT474. In addition, the proportion of phosphorylated DEPs in BT474 mostly exceeded BT474R (Figure 1d), alluding to a more active cell signaling state.

We utilized ATAC sequencing to establish the chromatin state, while transcriptional changes were assessed using RNA sequencing. Samples were prepared for ATAC and RNA sequencing, processed, and analyzed, as highlighted in the Section 2. The number of differentially accessible regions (DARs) is summarized in Figure 1e (Table S2 and Figure S1). Our results from ATACseq indicated a larger proportion of DARs in BT474, in contrast to BT474R, substantiating a more “active” cell state. While DARs in EGF-BT474R increase with time, EGF-BT474 was notably most accessible at the 12 h time point. Annotation of the DARs indicated that a higher proportion of these peaks were captured within the promoter regions of genes in BT474 compared to BT474R (Figure 1f). Many of the differential peaks identified were observed to be in the downstream and distal intergenic regions of known genes for both cell lines (Figure S2a,b). A preliminary analysis of genes that have significant accessibility in the promoter regions after treatment shows enrichment for functions such as androgen response, estrogen response in EGF-BT474 (adj *p* < 0.05), and inflammatory response and hypoxia in BT474R (adj *p* < 0.05) (Figure S2c,d). Differentially expressed genes (DEGs) were likewise calculated for each pairwise comparison (treatment vs. control) at every time point (1 h, 12 h, and 24 h) (Figure 1g, Table S3). Based on the number of DEGs, BT474 was more transcriptionally active than BT474R, consistent with our RPPA and ATACseq data. While the two treatments elicited a mostly linear increase in response across time in BT474R, the strongest transcriptional response in BT474 was at 12 h, consistent with DARs from ATACseq. The top 50 DEGs identified in each comparison are presented in Figure S3.

All the processed data were made available via an RShiny app at https://sc-bcwgweb.sdsc.edu/shiny/BT474_RShiny/, which offers the user flexibility to choose other conditions of interest in addition to what has been presented within this manuscript. The details on the RShiny app development are presented within the methods.

### 3.3. Plasma Membrane HER2 Clustering Using qSMLM

**Figure 2:**
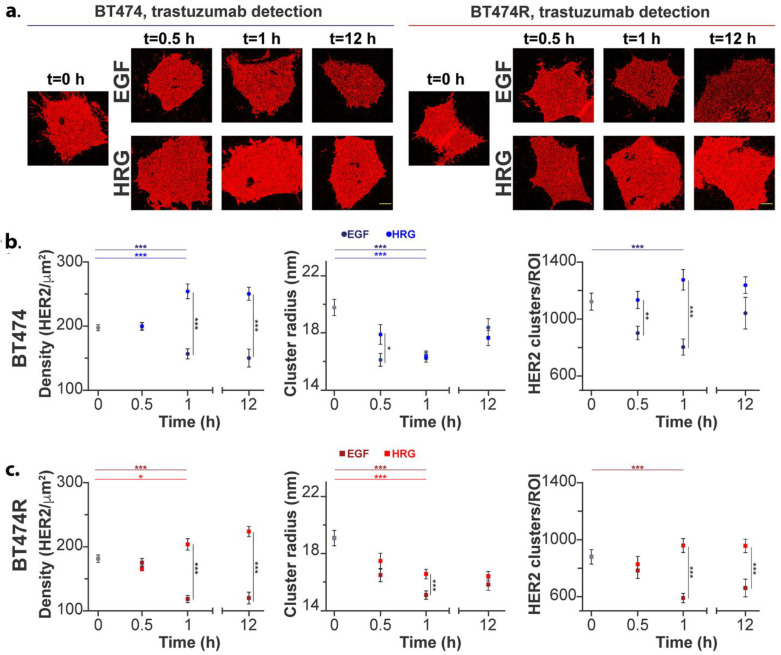
Membrane HER2 clustering, as detected through qSMLM in BT474 and BT474R after EGF and HRG treatment. (**a**) HER2 was detected using trastuzumab-AF647 as probe in BT474 and BT474R. Cells were imaged with SMLM after 14 h starvation (t = 0 h) or after 14 h starvation, followed by treatment with ligands for the indicated time. Red points are the (x,y) coordinates of localizations detected using SMLM in representative cells. Scale bars are 5 µm. (**b**,**c**) Average detected HER2 densities and clustering parameters after EGF treatment and HRG treatment are shown for each cell line (BT474 in (**b**) and BT474R in (**c**)). A total of 10–20 cells (36–77 ROIs) were analyzed for trastuzumab detection. Clustering parameters were calculated from clustered ROIs; the total number of these ROIs ranged from 19 to 53, depending on the ligand and time point. Graphs are plotted for each individual time point using the mean value. Error bars are represented by standard error of the mean (SEM). For clarity, only significant *p* values between EGF and HRG treatment for each cell are shown (*p* value indicated as * <0.05; ** <0.01; *** <0.001.); all other numerical details and additional clustering parameters are provided in Table S4.

HER2 activity is greatly influenced by the dimerization dynamics with EGFR and HER3/4, as well as its rates of endocytosis and recycling [6]. To better decipher the effect of HER2 activation and downstream signaling changes, we first sought to assess the molecular organization at the membrane using qSMLM. Using a trastuzumab-AF647 probe, we assessed HER2 organization in the starved state (T0) and after treatment with ligands EGF and HRG (see Section 2 for details on qSMLM). To enable robust molecular counting [9], we first determined the photophysical parameters of the fluorescent probe (trastuzumab-AF647) using MDA-MB-468 cells (Figure S4a). Next, using trastuzumab-AF647, we assessed detected HER2 densities and clustering parameters using pair correlation and k-means-like clustering analysis of the SMLM data [9,30,31]. Lateral localization precision distributions are shown in Figure S4b,c. At T0, we saw significantly higher detected HER2 densities in BT474 vs. BT474R (Figure 2 and Table S4). We also observed a significantly higher number of HER2 clusters and fraction of clustered HER2 in BT474 compared to BT474R cells (Table S4). These data are in agreement with our recent work performed in cell lines and patient specimens [8].

We next assessed the effect of ligands on HER2 organization using EGF and HRG as activation ligands. With EGF, we observed a significant reduction in detected HER2 densities at 1 h in both BT474 and BT474R (Figure 2b,c (left), Table S4). Detected HER2 densities remained largely unchanged at 12 h. However, with HRG, we observed a significant increase in detected HER2 densities at 1 h, which did not change appreciably at 12 h for both BT474 and BT474R. Additionally, higher detected HER2 densities were observed in BT474 compared to BT474R across time for HRG treatment. Both EGF and HRG treatments led to a significant decrease in cluster radii at 1 h (Figure 2b,c (center), Table S4). For both EGF and HRG treatment, recovery of cluster sizes toward the starved state level at 12 h was more pronounced in BT474 cells compared with BT474R cells. Next, in both BT474 and BT474R, we observed a reduction in the number of HER2 clusters after EGF at 1 h (Figure 2b,c (right), Table S4). At 12 h, values were trending toward starved-state levels, with a more pronounced effect in BT474. In both BT474 and BT474R, we observed a slight but not significant increase in the number of clusters after HRG (Table S4). Importantly, there was a significant difference between the two ligand treatments at 30 min and 1 h time points for BT474 and at 1 h and 12 h time points for BT474R (Table S4). At all three time points after HRG treatment, the number of HER2 clusters was higher in BT474 cells compared to BT474R cells.

### 3.4. Receptor Organization and the PI3K/AKT/mTORC1 Signaling

**Figure 3:**
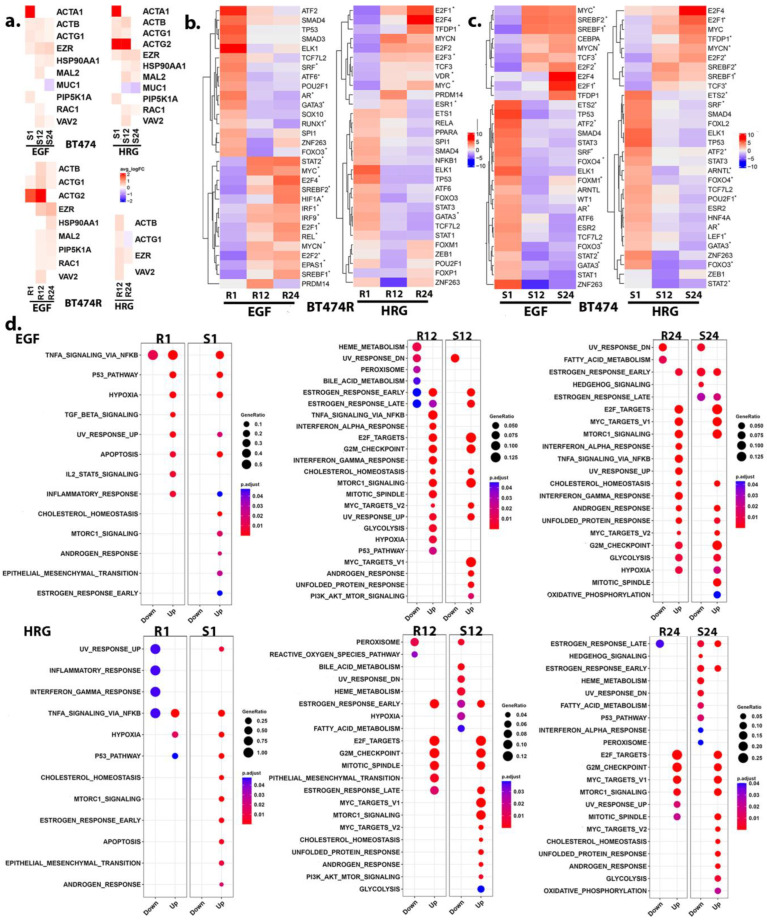
HER2 clustering and downstream signaling. (**a**) Heatmaps highlight the fold changes observed for genes that are known to affect HER2 organization at the membrane. HRG-treated BT474R shows an absence of significant regulation of several of the transcripts within our data, alluding to altered HER2 membrane organization. (**b**) Transcription factor (TF)-target activity, as ascertained by DoRothEA, highlights the increased activity of IRF TFs (red) and HIF1A in EGF-treated BT474R, particularly at the later time points. * indicate TF, which were also identified as DEGs at least one time point. (**c**) A similar DoRothEA TF-target activity heatmap for BT474 treated with EGF and HRG. (**d**) A comparative dotplot highlighting the Hallmark enrichment identified for BT474R (R1, R12, and R24) and BT474 (S1, S12, and S24) after EGF (upper panels) and HRG (lower panels) treatments. Both (**b**,**c**) consistently showed activation of PI3K/AKT signaling in BT474 (adj *p* < 10^−3^) and its downstream mTORC1, particularly at early time points.

Given the distinct differences in HER2 measurements between BT474 and BT474R, we next assessed the molecular mechanisms involved in HER2 activation and signaling. Several studies have highlighted the dynamics of HER2 membrane organization, retention, and internalization [6,32,33,34,35,36]. EGF-BT474 and HRG-BT474 showed transcriptional regulation of several proteins with known roles in HER2 organization, including *ACTB*, actinins, *RAC1*, *VAV2*, *HSP90*, and *MAL2* (especially significant at 12 h) (Figure 3a). This was also reflected in the RPPA data with ACTB (antibody name: b Actin), CAV1 (antibody name: caveolin 1), MUC1 (antibody name: EMA), and PAK1 (antibody name: PAK1) being significantly higher at more than one time point within EGF-BT474. While EGF-BT474R showed transcriptional regulation of several membrane proteins highlighted above, HRG-BT474R showed a distinct lack of regulation, alluding to more prominent alterations in HER2 organizational dynamics in BT474 and EGF-BT474R (with respect to untreated controls).

PI3K signaling and feedback serve as a crucial regulator of HER2 membrane dynamics. For instance, the recruitment of the HER2/VAV2/RAC1/PAK1/actin/actinin complex to cell protrusions and cell motility were abrogated by the inhibition of PI3K [33]. PI3K/AKT/mTORC1 signaling axis is known to be crucially affected downstream of HER2 [37,38]. RPPA data from early time points (30 min and 1 h) showed a robust and expected activation of PI3K/AKT/mTORC1 signaling [39] (see Section 2) in BT474 (Figure S5). Functional analyses of the transcriptome, including TF-target activity analyses (via DoRothEA) (Figure 3b,c) and functional enrichments (Figure 3d, Table S5), consistently showed a sustained activation, including PI3K/AKT/mTORC1 signaling in BT474 (adj *p* < 10^−3^), particularly at later time points after EGF treatment [40,41,42], consistent with transcriptomic measurements. Interestingly, we observed sustained activation of RICTOR and RICTOR_pT1135 within our RPPA data for EGF-BT474, suggestive of increased activity for the mTORC2, which could act in parallel with mTORC1-dependent feedback mechanisms affecting AKT signaling [43]. In contrast, BT474R cells did not show significant regulation of PI3K/AKT and canonical mTORC1 activation cascade, particularly after treatment with HRG. These were also reflected in the RPPA measurements (Figure S5).

### 3.5. Differential Bioenergetics in BT474 and BT474R

**Figure 4:**
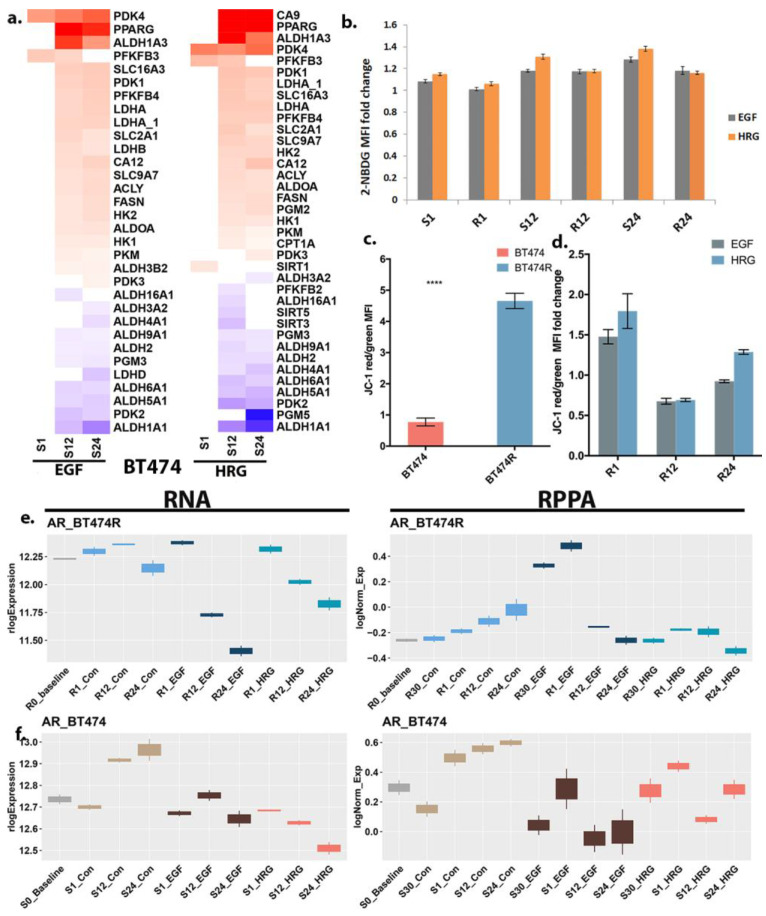
Differential bioenergetics in BT474 and BT474R. (**a**) Heatmaps highlight the fold changes observed in glycolysis-associated gene products in BT474, with both HRG and EGF-BT474, specifically at the later time points. (**b**) Glucose (2-NBDG) uptake in BT474R at different time points (R1, R12, and R24) and in BT474 (across time, S1, S12, and S24) after EGF and HRG treatment show a fold change over control. Flow cytometry analysis of serum-starved BT474 incubated with 2-NBDG after treatment for 1 h, 12 h, and 24 h. Error bars represent the SEM of three independent experiments (n = 9). (**c**) Active mitochondria exhibit brighter red fluorescence signal compared to mitochondria with lower membrane potential, which fluoresce green. Changes in the red/green fluorescence signal ratio can be used to determine healthy versus depolarized mitochondria (mitochondria membrane potential (Δψm)). Bar plot represents the Δψm (JC-1 red/green median fluorescence intensity (MFI)) of serum-starved BT474 and BT474R cells stained with MitoProbe JC-1 (*p* indicated as **** < 0.0001). (**d**) Bar plot shows the JC-1 red/green MFI after EGF and HRG treatment for 1 h, 12 h, and 24 h in BT474R (fold change over vehicle). Error bars represent the SEM of three independent experiments (n = 9). (**e**) RNA and RPPA measurements of AR across all treatment and time point combinations are shown for BT474R. (**f**) RNA and RPPA measurements of AR across all treatment and time point combinations are shown for BT474.

mTORC1 is a major regulator of cellular bioenergetics via glycolysis and a major nutrient/energy sensor. As noted earlier, BT474 (irrespective of treatment) dominantly showed mTORC1 activation [41], which drives the activation of TFs such as *HIF1A*. *HIF1A* is a master regulator of glycolysis and potentiates the transcription of glucose transporters and glycolytic enzymes, including *SLC2A1*(GLUT1), *HK2*, *PDK1-4*, and *PKM* [44,45], which were significantly upregulated in BT474 at multiple time points (Figure 4a, Tables S1 and S3). Other crucial players (both aerobic and anaerobic) in glycolysis, including *PFKP/PFKM*, *ALDH2*, *PGM*, *CA9/12*, *PPARGC1A/B*, and *SLC1A5*, were also dysregulated in BT474. Suppression of SIRT activity has been previously suggested to activate HIF1A-mediated glycolysis [46]. Our measurements showed suppression of SIRTs (*SIRT3* and *SIRT5*) and their upstream regulators, including *PPARA, FOXO1,* and *FOXM1* (both seen in RPPA and ATACseq, Supplementary Tables S1 and S2), prominently at later time points (Figure 4a). In line with RPPA and RNAseq measurements, BT474 showed an elevated glucose uptake, as detected via glucose-uptake assays, irrespective of treatment (Figure 4b) with respect to their controls, in contrast to BT474R.

While our enrichment results in BT474 captured an increase in OXPHOS compared to controls, BT474R showed inherently high levels of mitochondrial activity, irrespective of treatment, consistent with prior research [47,48], especially at the earliest time point (Figure 4c,d). In particular, omics measurements in EGF-BT474R showed a distinct activation of several genes such as *GATA6*, *SMAD4*, and *MYC* at early time points (30 min–1 h) (Tables S1 and S3). Activation of several genes/proteins highlighted above have been previously implicated in aerobic glycolysis (Warburg effect) and could drive the mTORC1 signaling seen at later time points in BT474R [49]. Several of these genes also had significant enrichment for open enhancers across both treatments and multiple time points (Table S6). Taken together, these allude to a state of flux between glycolytic and mitochondrial metabolism [28] in BT474 across time and a preferred early mitochondrial bioenergetic state in BT474R.

### 3.6. Androgen Response in BT474 and BT474R and Its Metabolic Implications in HRG-Treated BT474R

AR expression is known to be inherently high in the BT474 cell line [50]. Both RPPA and RNAseq data reflected high inherent AR expression in BT474 (control and baseline measurements. However, we observed an overall reduction in AR mRNA and protein expression after EGF and HRG treatments in both BT474 and BT474R (Figure 4e,f). Research in prostate cancer (PCa) cell lines has previously demonstrated a PI3K-independent suppression of *AR* mRNA after EGF ligand treatment and a PI3K-dependent mechanism for AR degradation after HRG treatment [51,52]. Although, to the best of our knowledge, no evidence exists in the literature for the effect of ligand treatment on AR expression in BT474R, we observed trends similar to BT474, albeit with some differences highlighted below.

AR gene and protein expression decreases with time for both EGF and HRG in BT474R. Notably, HRG-BT474R showed a more drastic reduction in AR protein, in contrast to AR mRNA, across time. Since androgens are known to modulate lipid metabolism in PCas by promoting the expression of sterol regulatory element-binding proteins (SREBPs) with direct binding sites in FASN [53], we wondered if there were any significant differences in lipid metabolism between HRG-BT474R and EGF-BT474R. Notably, while several of the SREBPs and other lipogenic genes were broadly dysregulated, HRG-BT474R showed a distinct lack of significant regulation of *SREBF1/2* targets and several downstream genes, including *HMGCR*, *NR1H3(LXR)*, *DSS*, and *FASN* (a crucial determinant of endocrine resistance) [54]. HRG-BT474R also showed no significant open enhancer (as detected through PEREGRINE analysis, Table S6; see Section 2) or binding regions for SREBF on its downstream targets (Table S2), suggestive of altered lipid metabolism and consequently an altered energy homeostatic state in HRG-BT474R, likely dependent on the early differential AR dynamics.

### 3.7. Activation of the IRF1 Cascade in EGF-Treated BT474R

**Figure 5:**
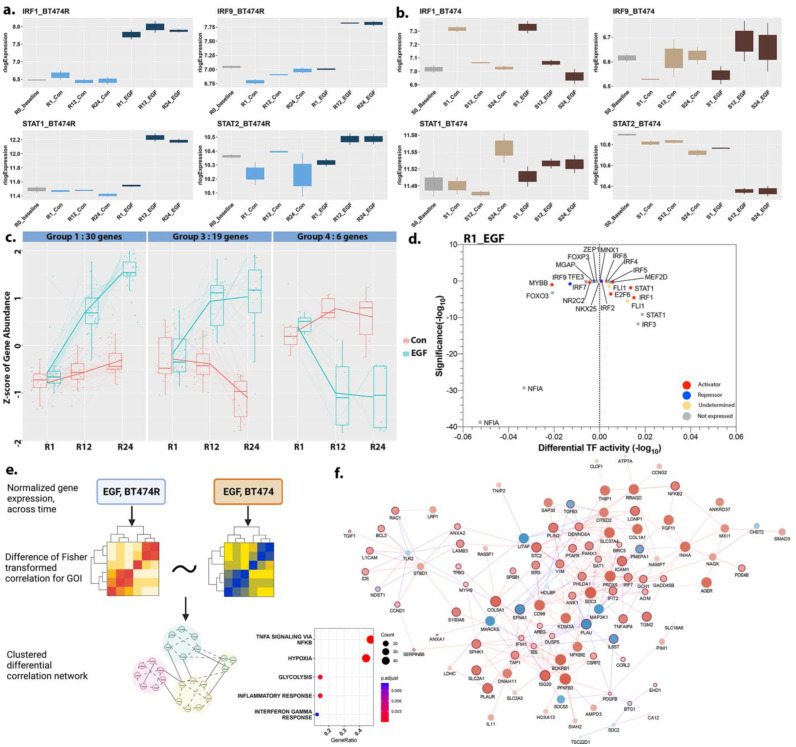
Differential signaling between EGF-BT474R and EGF-BT474. (**a**) Boxplots of four crucial TFs, *IRF1*, *IRF9*, *STAT1*, and *STAT2*, in BT474R and BT474, respectively, highlight the increase in expression after EGF treatment. (**b**) The differential dynamics of these four TFs in BT474 after EGF treatment. (**c**) Gene abundance signatures of 60 upstream and downstream interactors of IRF1 from custom EGF treatment-specific TF-target/PPI network (see Section 2) clustered into three groups via degPatterns showed a distinctly diverging profile across time for IRF1, STAT1, and associated proteins between EGF and controls in BT474R (also see Table S6). (**d**) Diff TF analysis for EGF-treated BT474R at 1 h highlighted the role of IRFs, particularly IRF1, as an activator. DiffTF results for 12 h and 24 h after EGF in BT474R are shown in Figure S5. (**e**) An infographic showing the derivation of differential correlation network for EGF-treated BT474R and BT474. Clustering is performed using a metric called topological overlap measure (see Section 2). (**f**) The turquoise module with the largest number of differential intramodular edges was identified as being differential between BT474R and BT474. The red edges highlight a positive correlation among genes such as *SDC3*, *CITED2*, *CD99*, *PFKFB3*, *PRDX5*, and *BDKRB1*. Notably, several of these genes have been previously implicated with significant roles in cancer progression and chemoresistance Note: for the edge colors, red indicates higher correlation in BT474R, and blue indicates higher correlation in BT474. For the node colors, red nodes are upregulated by fc value, blue nodes are downregulated by fc value, and green node names are associated with glycolysis. Inset—dotplot of enrichment of the nodes identified in this module.

EGF-BT474R uniquely displayed a distinct upregulation of gene cascades associated with response to interferons (IFN) α/γ (adj *p* < 10^−3^), particularly *IRF1* (Figure 5a). It has been hypothesized that BT474 exhibits constitutive activation of *IRF1* [55]. Despite the unstable and short half-life of the IRF1 protein [56], we also observed increased IRF1 protein within the RPPA data (Table S2, with adj *p* < 0.05 only at 24 h). Upon stimulation with EGF, *EGFR* activity is known to induce IRF1 gene cascades, largely through STAT1 phosphorylation/activation, in other cancer cell lines [57]. Sequencing data from BT474R after EGF treatment likewise showed an early increase for IRF1. *IRF1* expression in certain animal models is shown to precede and influence the expression of other interferon-stimulated genes (ISGs), including *STAT1*, *STAT2*, and *IRF9* [58,59]. These four proteins together have been predicted to form a positive feedback amplifier circuit regulating the IFN response [60]. *IRF9*, *STAT1*, and *STAT2* all showed increased activity at 12 h and 24 h, especially in BT474R, in contrast to what is observed in BT474 (Figure 5a,b). We further assessed the impact of IRF1 signaling by extracting 60 upstream and downstream immediate interactors of IRF1 from the custom EGF treatment-specific TF-target/PPI network (see Section 2). Gene abundance signatures and clustering of these 60 interactors (using “degPatterns” in DEGreport [24]) showed a distinctly diverging profile across time for IRF1 and STAT1 between treatment and controls in BT474R (Figure 5c, Table S7). There were several interferon response genes downstream of *STAT1/IRF1* (group 1, Table S7), including *ISG15*, *IFI6*, *HDAC1*, *GBP2*, *CXCR4*, *CASP8*, *SLC26A6*, *TNFSF10*, *CXCL8* (a transactivator of *EGFR* [61]), and *OAS2/3*. Similarly, antigen-processing machinery (including *ERAP2*, *TAP1*, and *TAP2*) was also linearly increased across time upon EGF treatment in BT474R (group 3, Table S7). Several of the genes highlighted above were highly differentially expressed in BT474R and showed increased accessibility (adj *p* < 0.05) in their promoter region, as detected via DiffBind within BT474R after EGF, especially at >12 h. DiffTF analysis (which integrates ATACseq and RNAseq measurements; see Section 2) additionally highlighted IRF1 as a potent activator, especially at the earliest time point after EGF treatment (Figure 5d and Figure S6). Increased activity of downstream phospholipase C and other kinases could contribute to increased activity of NF-κB [62,63], further emphasizing a link between IRF1 driven NF-κB signaling in EGF-BT474R. In the following section, we evaluate the differences between BT474 and BT474R after EGF treatment in the context of NF-κB /HIF1A signaling.

### 3.8. A Comparison of Hypoxic Stress Response between BT474R and BT474 after EGF

Accumulating data from several studies indicate significant crosstalk between the HIF1A and NF-κB signaling pathways. HIF1A has been suggested to regulate NF-κB signaling through upregulation of p65 and IKKα. NF-κB is also able to bind the HIF-1A promoter and induce its expression [64]. Both BT474 and BT474R, compared to controls, showed evidence of hypoxic stress and NF-κB activation, albeit of differing magnitude. While BT474 (irrespective of treatment) demonstrated a HIF1A- and NF-κB -mediated hypoxic stress response, only EGF-BT474R showed a significant response across time (Figure 3b,d). Given this, we focused exclusively on EGF treatment to elucidate the nuanced differences in hypoxic response to treatment between the two cell lines. We utilized a differential co-expression network to ascertain functional differences between the response of the two cell lines.

A list of 515 genes known to be associated with hypoxia and NF-κB signaling was first compiled from resources (see Section 2, Table S8). A differential correlation network was constructed using the 385/515 present in EGF-treated BT474 and BT474R (see Section 2), resulting in a differential network of 341 nodes with 2980 significant edges (Figure 5e, Table S9). Clustering using the topological overlap measure (see Section 2) revealed the presence of three distinct modules after EGF treatment. The largest module (n = 148) also captured the largest number of intramodular edges that were significantly differential (adj *p* < 0.05, Figure 5f, Table S10) between BT474R and BT474. A detailed analysis of the largest connected component within the turquoise module indicated several genes (hubs detected; see Section 2) with increased correlation in EGF-BT474R and included *SDC3*, *CITED2*, *CD99*, *PFKFB3*, *PRDX5*, and *BDKRB1*. Particularly striking was the largest difference in genes that impacted glycolysis (Figure 5f, inset) and TGF-β signaling (*SDC2*, *SMAD3*, and *CITED2*) [65]. Several of these genes have been previously implicated with significant roles in cancer progression and chemoresistance. CITED2 is a crucial mediator of the HIF1A hypoxic response, which, in cancer, is indicated to serve as a co-activator of SMAD2/3 affecting TGF-β signaling [66]. CD99 and PFKFB3 have been previously found to mediate metabolic reprogramming, chemoresistance, metastasis, and stemness in ovarian cancer, likely via the modulation of inhibitors of apoptotic proteins and the NF-κB signaling pathway [67]. PFKRB3 is also a major target of EGFR signaling mediating glucose metabolism. While we did not identify statistically significant differential binding of these proteins, we observed active and open enhancer regions upregulated after EGF for several of these genes as analyzed by PEREGRINE, including *PFKFB3*, *SDC3*, and *BDKRB1*, especially at the later time points (Table S6). The increased correlation of the aforementioned genes in BT474R, compared to BT474, further alludes to a complex and differential interplay in the dynamics between hypoxia, NF-κB signaling, and metabolic reprogramming after EGF treatment between the two cell lines.

## 4. Discussion

The analyses presented here, to the best of our knowledge, present the most comprehensive molecular characterization of BT474R and BT474 cells in response to treatment with EGF and HRG. Our results indicate that EGF elicits a more robust response in BT474, as measured through its protein and transcriptional response and chromatin modification and alludes to it being a dominant driver of cell fate determination in these cell lines. The addition of EGF and HRG (compared to controls) activates cell cycle-associated processes prominently at later time points (e.g., E2F targets and G2M checkpoints) and increases the expression of transcription factors, including *MYC* and E2Fs (Figure 3b,c). This points to the propensity of tumorigenesis in the presence of endogenous ligands.

The qSMLM measurements indicate that EGF treatment leads to a significant reduction in the detected average HER2 densities and the number of HER2 clusters at 1 h in both BT474 and BT474R, in contrast to HRG treatment, which shows increased HER2 densities, specifically at 1 h (Figure 2). The reduction in cluster radii at 1 h after EGF and HRG further alludes to the tight packing of HER2 clusters. Omics measurements of total HER2 (especially up to 12 h), however, show no decrease compared to the control or baseline (Figure S7). As noted earlier, several factors can influence the observed HER2 membrane dynamics and its internalization or trafficking within the cell. Endocytosis of HER2 can occur through clathrin-mediated or caveolin-mediated pathways, and internalized receptors can be recycled back to the membrane or targeted for degradation. In addition, HER2′s heterodimerization dynamics and the intracellular Ca^2+^ concentration [68,69] can affect its localization and activity. The analysis of omics measurements shows significant alterations in EGF-treated BT474 and BT474R (with respect to control), and intracellular Ca^2+^ dynamics indicate a more significant shift after EGF treatment over HRG (Figure S7). Taken together, these findings allude to significant alterations in membrane dynamics after EGF, which can be attributed to higher HER2 endocytosis or active HER2 reorganization that persists and leads to increased steric effects and is worth further exploration. The PI3K/AKT/mTORC1 signaling axis is known to be crucially affected downstream of HER2 [37,38]. BT474R, particularly after HRG treatment, did not show significant regulation of PI3K/AKT/mTORC1 signaling or HER2 membrane organization-associated gene changes. Mechanisms associated with increased HER2 membrane dynamics (as seen via qSMLM) contributing to the observed HER2 levels (Figure S7) and a reduction in the downstream signaling (as seen via omics measurements) compared to its respective controls after treatments warrants further targeted investigation.

Treated BT474 cells exhibited increased glycolysis (with respect to controls), as determined through the protein, gene expression signatures, and glucose-uptake assays. BT474R, consistent with our current understanding, shows evidence of inherently higher mitochondrial activity in its starved state (Figure 4c). However, notable differences in response to HRG treatment exist in BT474R. In particular, HRG-BT474R shows a considerable reduction in *AR* expression and *AR* response gene activity, compared to untreated controls, in contrast to BT474 with respect to untreated controls. Androgens are known regulators of several lipid metabolic pathways in PCa, including lipid uptake, biosynthesis, and degradation. The AR directly (via binding to the androgen response element) or indirectly (via activation of SREBP) regulates key lipid metabolic genes. Free fatty acids (FFAs) are known to serve as dominant bioenergetic substrates that feed into the tricarboxylic acid (TCA) cycle for energy production (increased OXPHOS) in cancer. EGF-BT474R, in contrast, shows a more dominant regulation of several genes/proteins associated with OXPHOS, including *SLC2A1*, *LDHA*, *SLC1A5* (ASCT2), and *SLC38A2* (SNAT2), in addition to genes/proteins associated with glycolysis.

Differential co-expression network analyses further highlight nuanced differences between EGF-treated BT474R and BT474, including an increase in TGF-β signaling in BT474R. This is consistent with the observed activation of SMAD targets, especially at the earliest time point in EGF-BT474R. TGF-β signaling is known to influence the bioenergetic dynamics within cancer cells (glycolysis, oxphos, and lipid metabolism) [70] and plays a role in PI3K activation (via TGF-β and HER2 interactions), contributing to trastuzumab resistance [71]. Increased activity of *CXCL8* (activated by NF-κB) and its neighbors is also observed in the differential network within EGF-BT474R. Numerous studies have confirmed that *CXCL8* can induce the phosphorylation of protein tyrosine kinases, including FAK and Src kinases, driving metabolic changes [63,72,73]. The contribution of microenvironmental hypoxia to NFκB-induced response to cellular stress is being increasingly appreciated [64]. HIF1A and NF-κB can co-operatively activate genes involved in tumor growth, progression and metastasis, metabolic reprogramming, and resistance to chemotherapy [74,75]. Taken together, these results highlight a temporal fluidity in the bioenergetic state and its largely treatment-dependent interaction with HIF1A/NF-κB signaling in BT474R.

Previous studies in solid tumors have demonstrated that prolonged interferon signaling allows tumors to acquire STAT1-related epigenomic/genomic changes contributing to the maintenance of resistance to immune checkpoint blockades [76], with a major contributor of IFNG being tumor-associated T cells. The prolonged and sustained activation of the IRF1/STAT1 cascade in the EGF-treated BT474R alludes to an interferon gamma-independent mechanism likely mediated by EGFR that contributes to the maintenance of resistance through the IRF1 cascades. Though we were unable to capture the precise transcription to translation dynamics of *IRF1*, our multiomics data point to potent and sustained activation of *IRF1* and its downstream targets and warrant further investigation. IRF1 is a regulator of diverse cellular functions and a known tumor suppressor whose endocrine responsiveness regulates cell fate in cancers [77,78]. IRF1, as seen in our data, could impact several downstream processes, including the antigen-processing machinery, activation of CXCL8 and Phospholipase C, and subsequent NF-κB activation [62,63], several feedback mechanisms contributing to EGFR transactivation and consequently the sustained activation of IRF1/STAT1 signaling.

## 5. Conclusions

**Figure 6:**
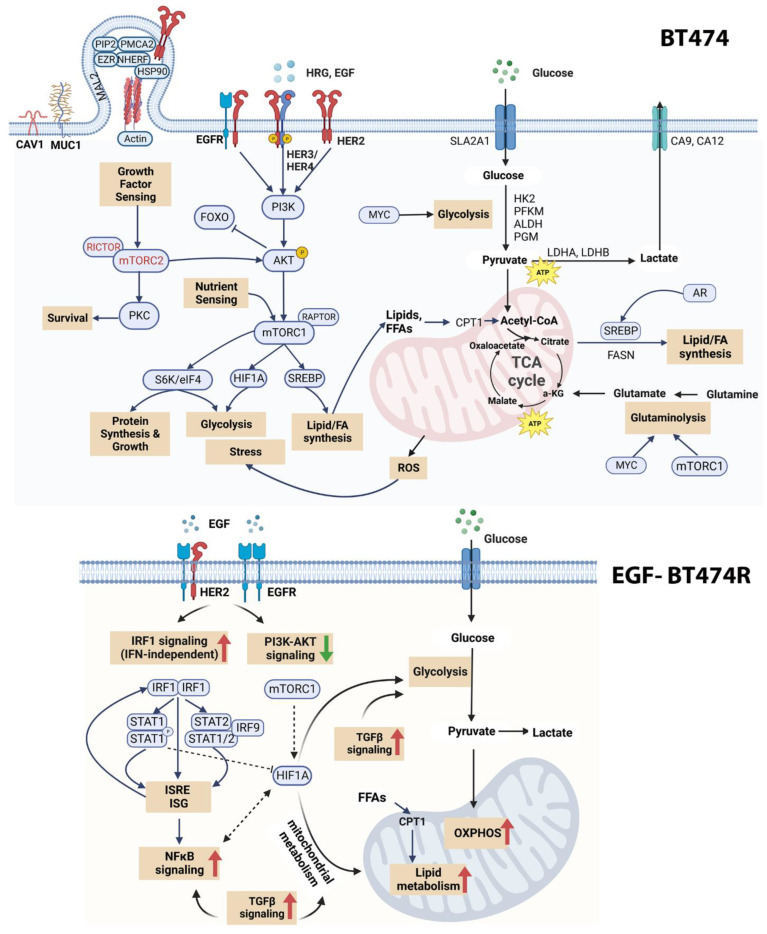
Mechanistic underpinnings of BT474 and BT474R. The top panel represents the major mechanisms observed via our multimodal measurements as being prominent in BT474, irrespective of treatment. We observed an active PI3K/AKT/mTORC1 cascade, evidence of both aerobic and anaerobic glycolysis, increased lipogenesis mediated by AR, and HIF1A-mediated stress response, which also contributes to the metabolic reprogramming in BT474. BT474 shows a mix of substrate utilization for bioenergetics across time and treatments. The bottom panel captures the findings of this study as it pertains to BT474R, particularly after treatment with EGF. BT474R showed inherently high mitochondrial bioenergetics for both ligands but showed a ligand-dependent activation/absence of certain signaling cascades. Only EGF-BT474R displayed increased IFN-independent, IRF1-mediated signaling, which has been reported to be a consequence of EGFR activation. The self-amplification circuit using STAT2, IRFF9, and STAT1 likely contributes to the sustained IRF1 activation across time. The downstream activation of interferon-stimulated genes (ISGs) and interferon-sensitive response element genes could impact cellular bioenergetics through their activation of other signaling cascades, such as NF-κB. STAT1 repression of HIF1A could further contribute to the “flux state” in cellular bioenergetics within EGF-BT474R. As evidenced through our differential co-expression network analysis, an increased correlation of genes in EGF-BT474R associated with hypoxia, NF-κB signaling, and metabolic reprogramming further alludes to the differential bioenergetics with respect to BT474. HRG-BT474R (with respect to control) showed a distinct lack of FASN-mediated lipid metabolism-associated genes, likely mediated by early AR dynamics, and warrants further research.

Figure 6 summarizes the overall molecular underpinnings highlighted in this study. Our investigation studied the effect of ligand treatment on downstream mechanisms in BT474 and BT474R. While our study did not explicitly capture the combined dynamics of the endogenous ligands, it captures the role of EGF as a major determinant driving cellular dynamics. In particular, EGF-treated BT474R shows increased IRF1 signaling, which could be a direct consequence of EGFR activation. The self-amplification circuit of *STAT1/2* and *IRF9* likely contributes to the IFN1 independent and sustained activation of *IRF1* across time. Plasticity in metabolic regulation emerges from our study as a key factor in differentiating sensitive and resistant cells after treatment with ligands. HRG-treated BT474R shows altered FASN-mediated lipid metabolism, likely a consequence of reduced AR dynamics. While the mechanisms observed above do not highlight the origins of trastuzumab resistance, they provide insights into the potential consequence of acquired resistance, posing the intriguing question of whether addressing these altered mechanisms has the potential to reverse trastuzumab resistance. In summary, the findings described in this study provide avenues for drug development in cases where women acquire resistance over the course of trastuzumab treatment.

## Data Availability

Raw bulk RNAseq and ATACseq data have been submitted to the National Center for Biotechnology Information Gene Expression Omnibus under accession number GEO: GSE237607. All processed data, including the RPPA data, are made publicly available in the Supplementary Materials and through a publicly accessible RShiny server at https://www.tnbcworkbench.org/shiny/BT474_RShiny/. All code was written in R from established R/Bioconductor packages in line with their vignettes, and any information required to reanalyze the data reported in this paper is available from the lead contact upon request.

## Supplementary Materials

The following supporting information can be downloaded at: https://www.mdpi.com/article/10.3390/cancers16030553/s1, Figure S1: Binding affinities captured for each treatment condition, at every timepoint with respect to its control. Figure S2: Chromatin accessibility and enrichment. Figure S3. Fold change heatmap of all differentially expressed genes identified for each pairwise comparison of treatment vs control, at every timepoint. Figure S4. Photophysical properties of fluorescent probes and distribution of lateral localization precisions. Figure S5. Fold change heatmap of PI3K/AKT/mTORC1 pathway associated differentially expressed proteins and genes. Figure S6. Differential TF activity and accessibility Figure S7. HER2 expression and intracellular Ca^2+^ measurements. Table S1. Differentially expressed proteins detected from RPPA measurements for EGF and HRG treated BT474 and BT474R. Table S2. Summary of ATAC seq measurements captured for each cell lines, at each time point for both EGF and HRG. Table S3. Differentially expressed genes as detected via RNAseq for both cell lines, treated with EGF and HRG across time. Table S4: Summary of cluster measurements and associated *p* values with trastuzumab-AF647 as probe, for EGF and HRG treated cells. Table S5. Hallmark gene set enrichment of differentially expressed genes, across time, for both cell lines treated with EGF and HRG. Table S6: Enhancers peaks in EGF treated BT474R an. HRG treated BT474R as seen across time. Table S7: 60 interactors identified for upstream and downstream of IRF1 with clusters 1–4 identified through DEG pattern analysis. Table S8: A compendium of genes associated with Hypoxia and NFKB signaling, compiled as outlined in methods. Table S9. The differential network between EGF-BT474R and EGF-BT474, where each row represents an edge, between two gene pairs, and the corresponding *p* value of co-expression significance. Calculated as outlined in methods. Table S10. Highlights the nodes identified in the turquoise module within the differential co-expression network between EGF-BT474R and EGF-BT474.
