# Supplementary material for: Molecular Atlas of HER2+ Breast Cancer Cells Treated with Endogenous Ligands: Temporal Insights into Mechanisms of Trastuzumab Resistance
Source: Cancers (Basel). 2024 Jan 27;16(3):553. doi: 10.3390/cancers16030553 (PMC10854992; doi:10.3390/cancers16030553)
Supplement: Supplementary file 1 [file cancers-16-00553-s001.zip › AdditionalFile2 _postRevision.pdf]

Supplementary Figures

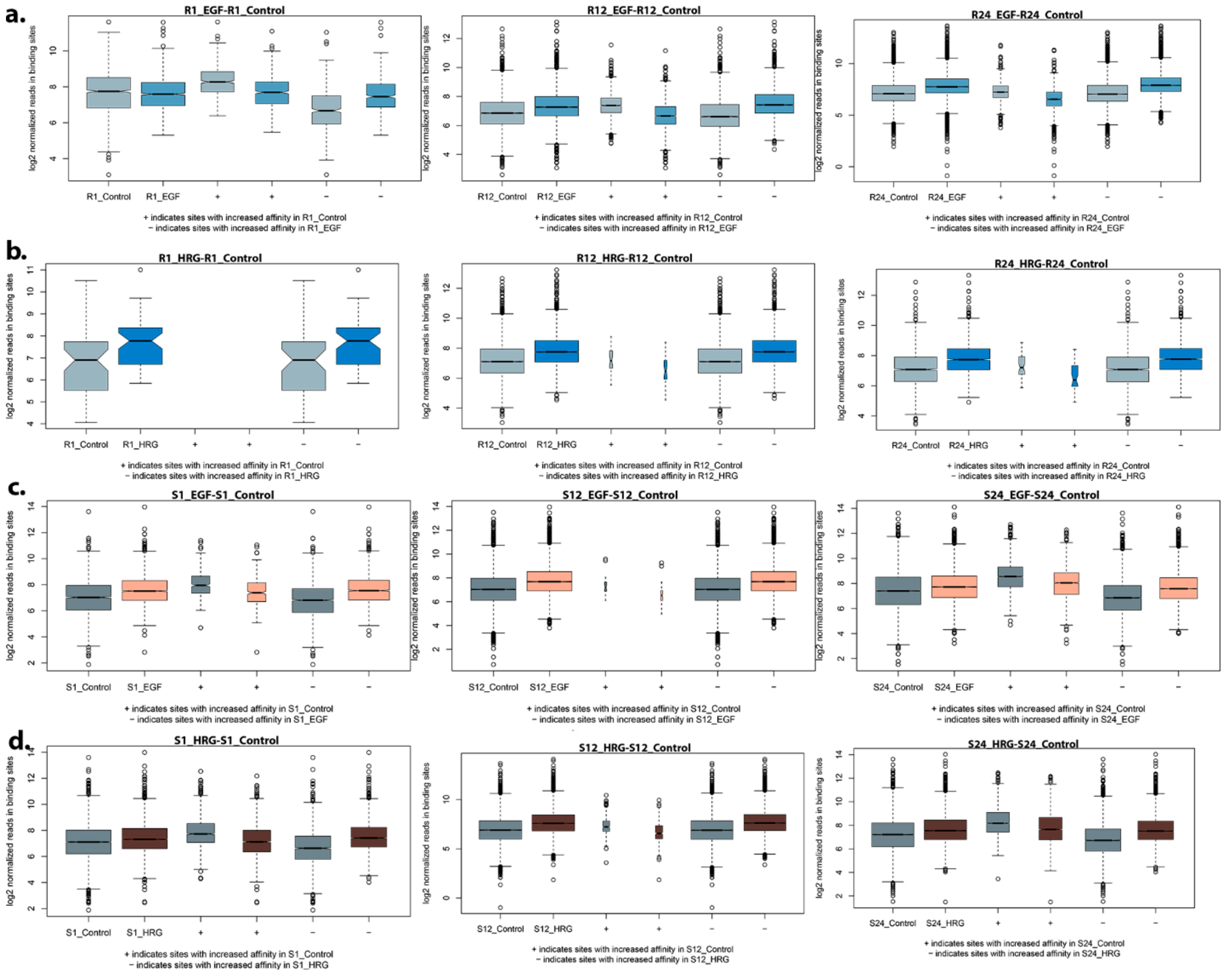

Figure S1: Binding affinities captured for each treatment condition, at every timepoint with respect to its control. *The first two boxes in every plot show the distribution of reads over all differentially bound sites in each group pair. The next two boxes show the distribution of reads in differentially bound sites that exhibit increased affinity in the control samples, while the final two boxes show the distribution of reads in differentially bound sites that exhibit increased affinity in the treatment samples at every timepoint.*

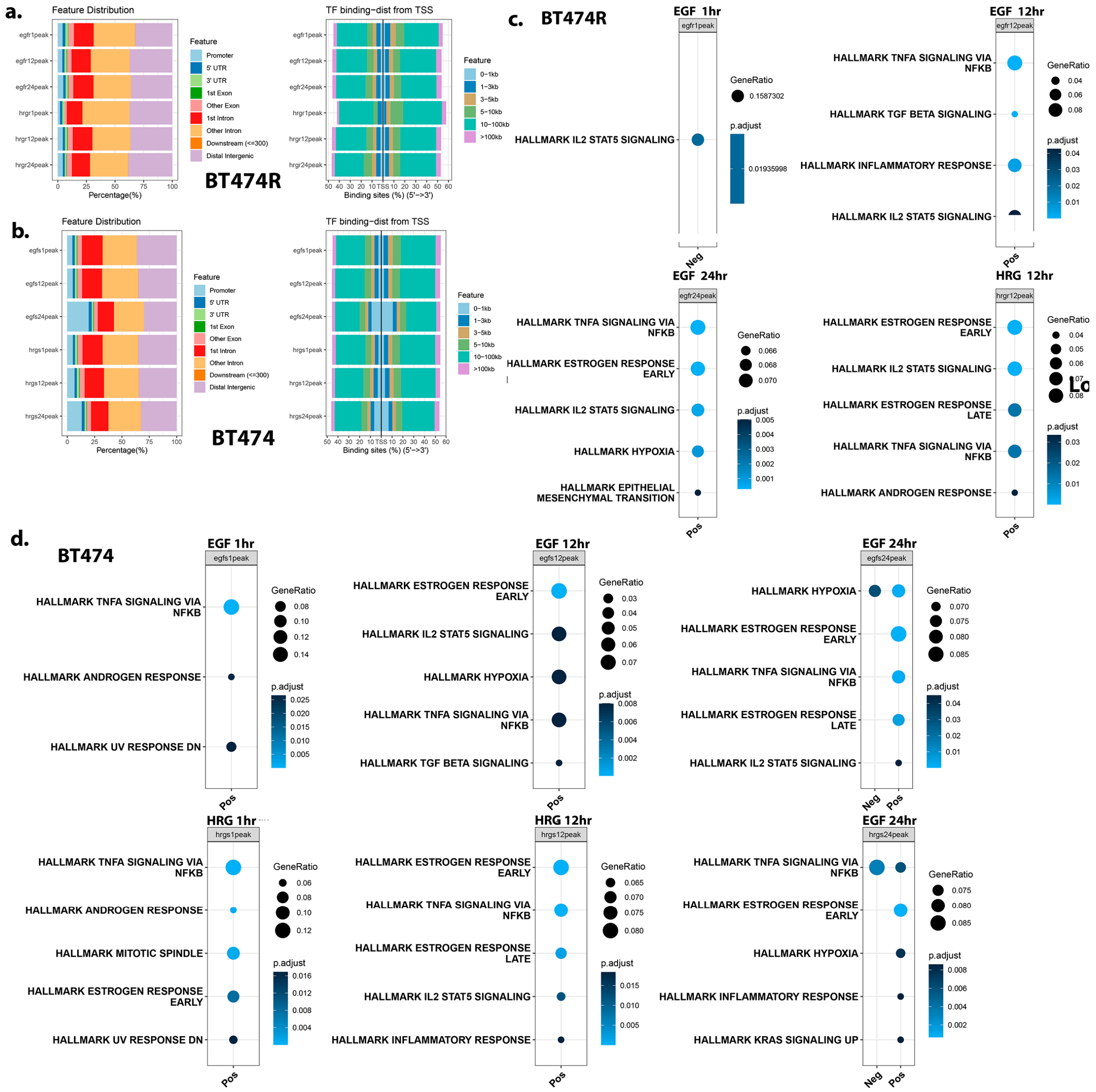

Figure S2: Chromatin accessibility and enrichment. *A. (left panel) Distribution of accessible peaks in BT474R after each treatment at every timepoint (Right panel) shows the percent of TF binding sites captured upstream and downstream of the transcription start site (TSS). B. Similarly shows the distribution of accessible peaks and distribution of the peaks from TSS in BT474. C. and D. Captures the hallmark enrichment of genes with significant accessibility in the promoter region for BT474R and BT474 respectively after every treatment at each timepoint.*

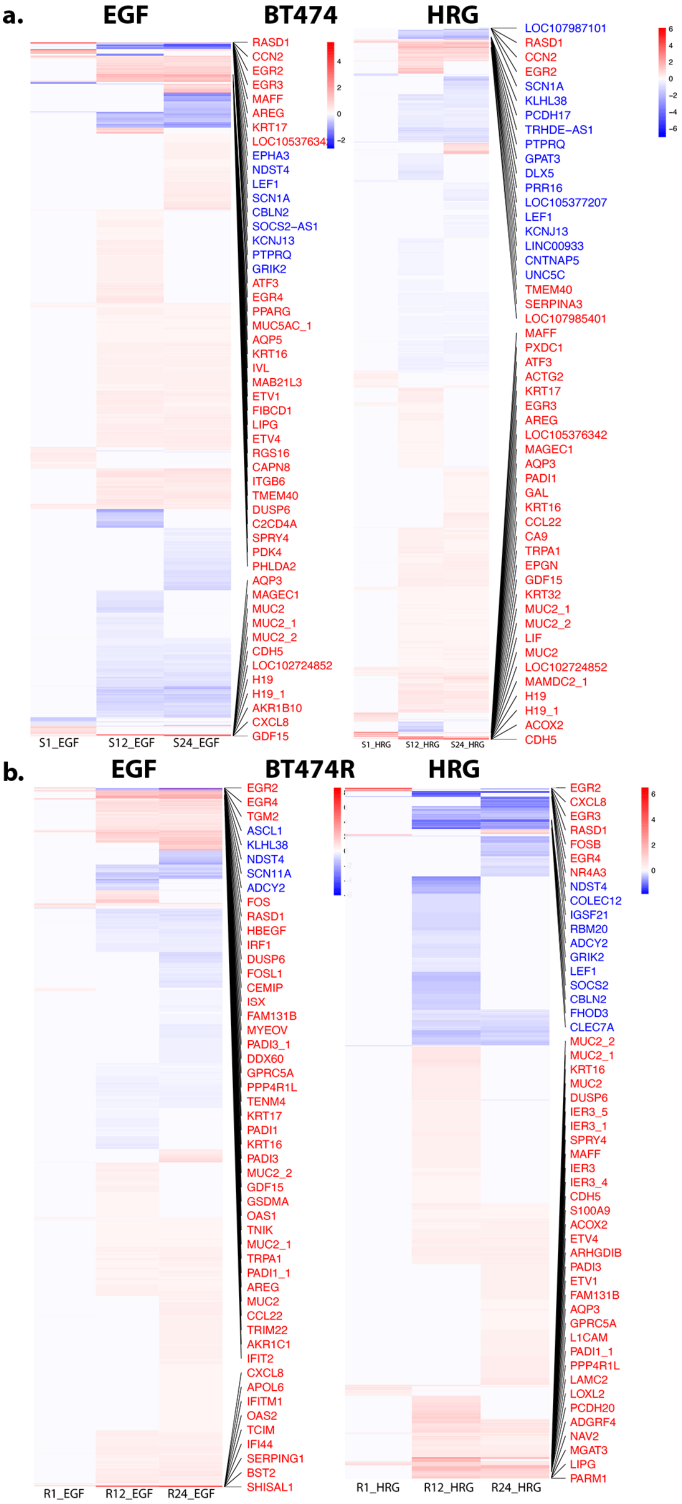

Figure S3. Fold change heatmap of all differentially expressed genes identified for each pairwise comparison of treatment vs control, at every timepoint. *A. (left panel) Highlights the names of top 50 DEGs identified after EGF treatment in BT474. (right panel) Highlights the names of top 50 DEGs identified after HRG treatment in BT474. B. Similarly represents the top 50 DEGs for EGF-BT474R (left panel) and HRG-BT474R (right panel).*

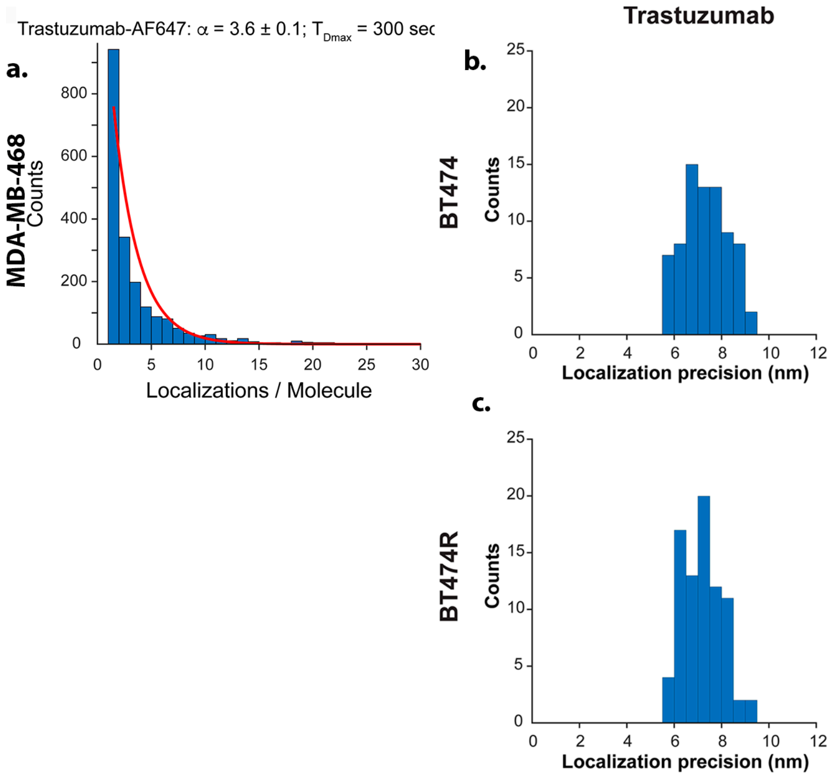

Figure S4. Photophysical properties of fluorescent probes and distribution of lateral localization precisions. a. *The average number of appearances for trastuzumab-AF647 was 4; the maximum dark time (T_{Dmax}) for probe was 300 s. MDA-MB-468 cell lines (starved for 14 h) were stained with 10 nM trastuzumab-AF647 to obtain sparse signal. Localizations were detected with a 64 µm² ROI as described before[37,39]. ROIs (13) were assessed across three separate experiments (n=3). b. Lateral localization precisions (σ) were obtained from NIS Elements software for all analyzed ROIs. Each analyzed ROI contributed an average lateral localization precision value to the overall distribution. The average σ from the distribution for BT474 cells: 7.3 nm for trastuzumab and c. 7.1 for trastuzumab in BT474R.*

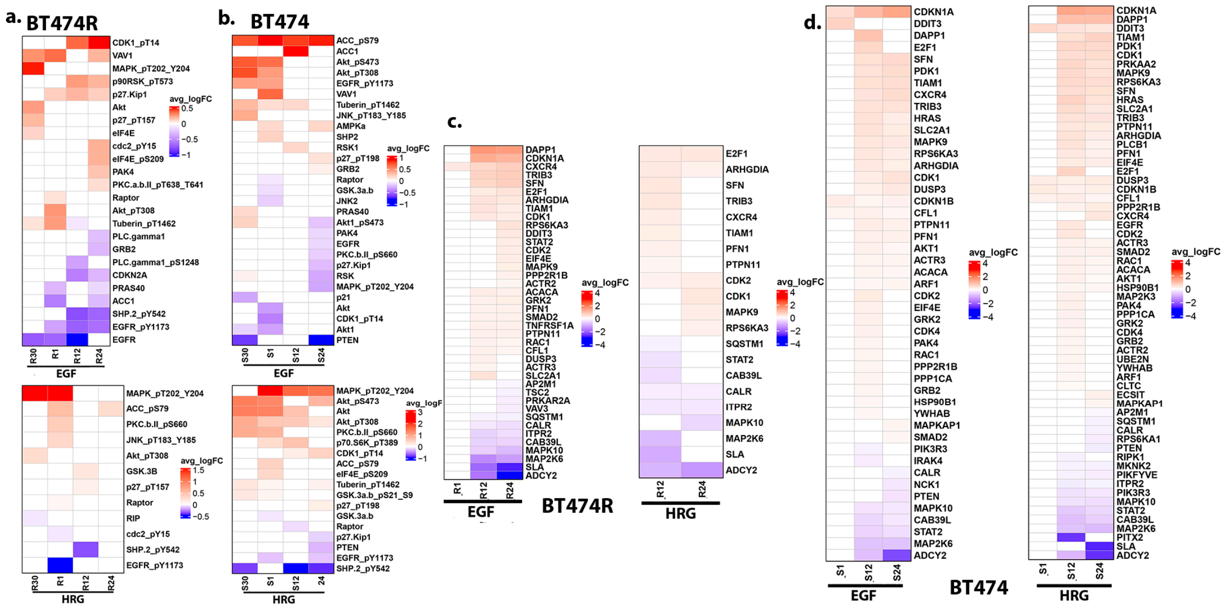

Figure S5. Fold change heatmap of PI3K/AKT/mTORC1 ***pathway associated differentially expressed proteins and genes. a****. DEPs in the Pi3k-AKT-mTOR pathway (from mSigDB) having significant regulation in EGF-BT474R (top) and HRG-BT474R (bottom) b. DEPs in the Pi3k-AKT-mTOR pathway having significant regulation in EGF-BT474 (top) and HRG-BT474 (bottom) c. DEGs identified in EGF-BT474R (left) and HRG-BT474R (right) in the* PI3K/AKT/mTORC1 *pathway. HRG-BT474R shows a distinctly diminished* PI3K/AKT/mTORC1 *response at a transcriptomic level. d. DEGs identified in EGF-BT474 (left) and HRG-BT474 (right) in the Pi3k-AKT-mTOR pathway*

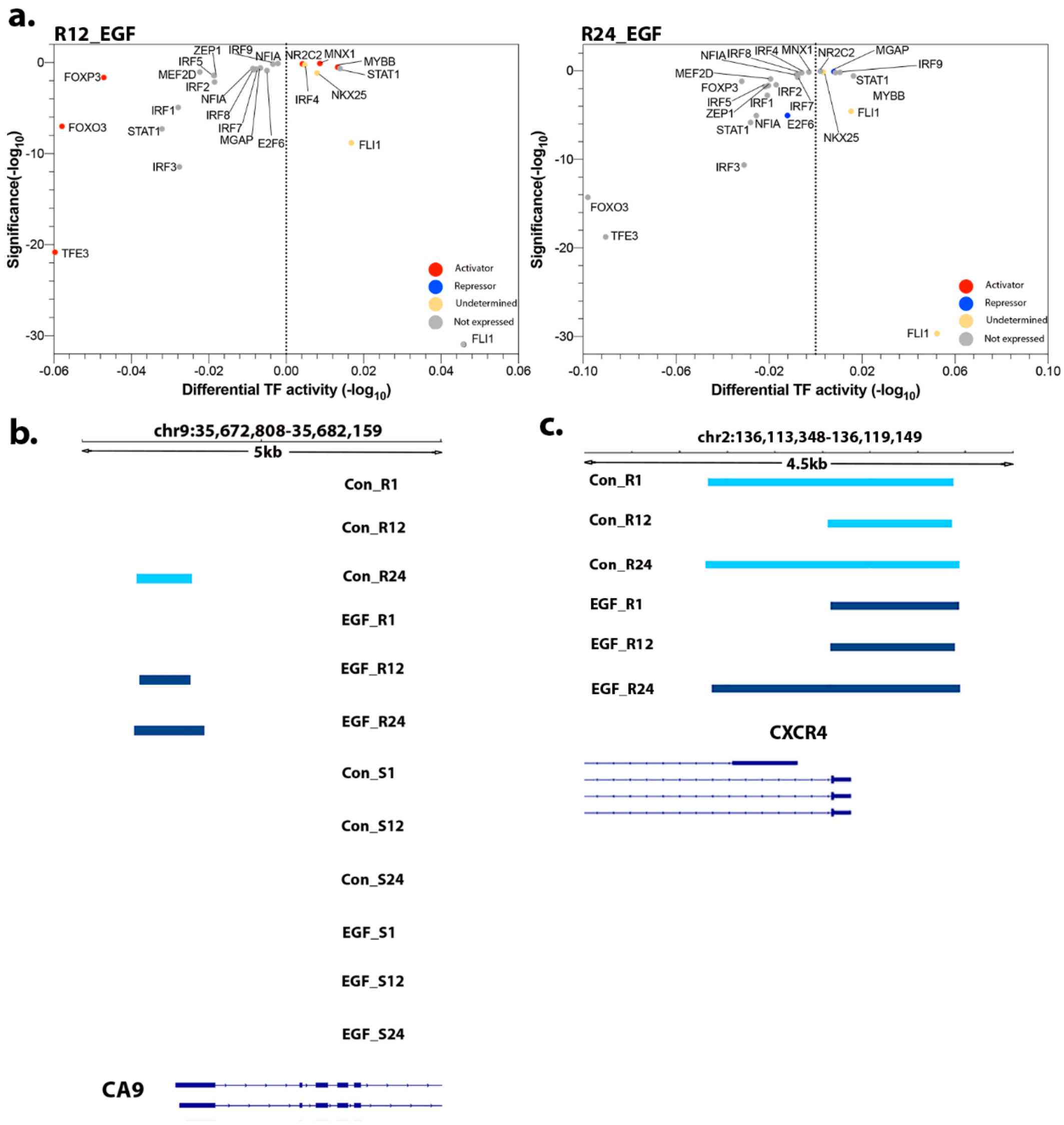

Figure S6 *a. DiffTF results shown for EGF-BT474R at 12 and 24 h respectively. We see significant activity for IRF1 associated signaling and associated TFs as outlined in the main text. b. Significant difference in consensus peaks identified in the promoter region of CXCR4 after EGF in BT474R. c . CA9, a highly sensitive endogenous sensor for hypoxic stress was found to be significantly transcriptionally upregulated as well as have significant differential binding (open) (p adj<0.05) in BT474R alone, after EGF treatment as seen by the presence of consensus peaks (for open regions) in EGF treated BT474R.*

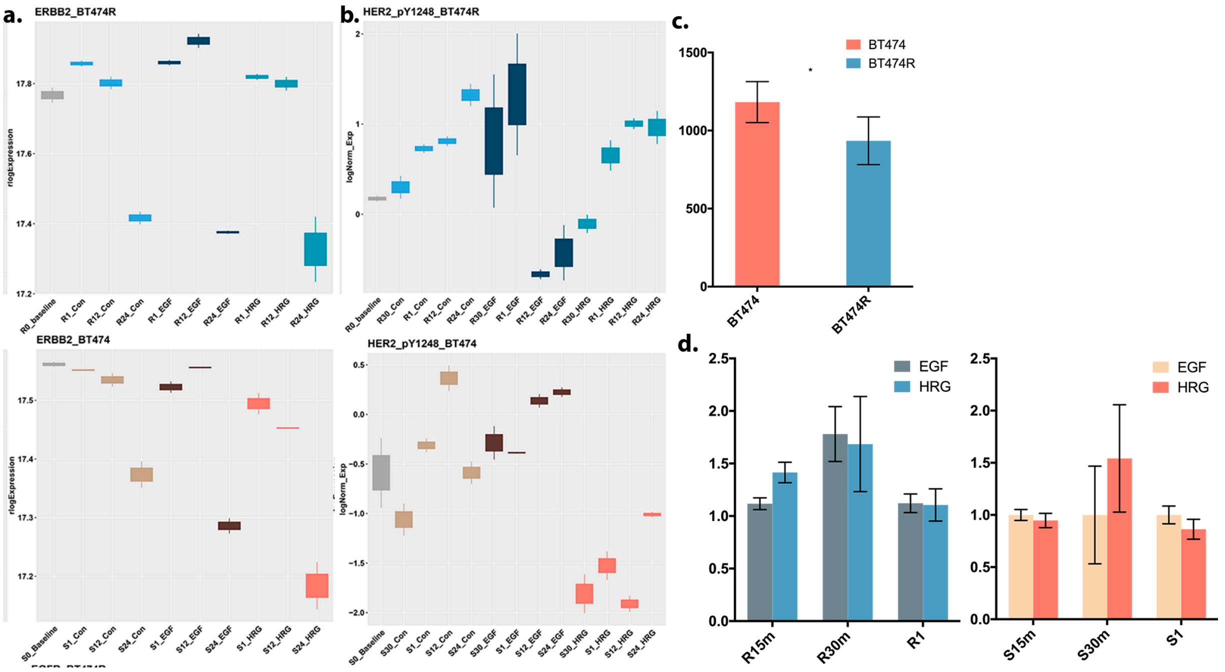

Figure S7 *a. HER2 expression (top panel) in RNAseq data for BT474R and BT474 (bottom panel). b. pHER2 expression as seen in RPPA data for BT474R (top panel) and BT474 (bottom panel) .c Intracellular Ca^{2+} measurements using Fluo-4 Direct in BT474 and BT474R after EGF and HRG after serum starvation. The mean fluorescence intensity (MFI) of a representative experiment is shown. Error bars represent SEM of technical replicates (n=3). d. MFI for BT474 and BT474R (right panel) cells treated with EGF and HRG for 15 min, 30 min and 1 h and loaded with Fluo-4 Direct for 1 h. The mean fluorescence intensity is shown as fold over vehicle treated controls. Error bars represent the SEM of three independent experiments (n=10).*

.
